# Benzene and 2-ethyl-phthalate induce proliferation in normal rat pituitary cells

**DOI:** 10.1007/s11102-016-0777-3

**Published:** 2016-11-16

**Authors:** Laura Tapella, Antonella Sesta, Maria Francesca Cassarino, Valentina Zunino, Maria Graziella Catalano, Francesca Pecori Giraldi

**Affiliations:** 1 Department of Clinical Sciences and Community Health, University of Milan, Milan, Italy; 2 Neuroendocrinology Research Laboratory, Istituto Auxologico Italiano, Via Zucchi 18, 20095 Cusano Milanino, MI Italy; 3 Unit of Oncological Endocrinology, Azienda Ospedaliera Universitaria Città della Salute e della Scienza di Torino, Turin, Italy; 4 Department of Medical Sciences, University of Turin, Turin, Italy

**Keywords:** Endocrine disruptor, Pituitary adenoma, Proliferation, Aryl hydrocarbon receptor (AhR), Aryl hydrocarbon receptor-interacting protein (AIP)

## Abstract

**Purpose:**

Endocrine disruptors are known to modulate a variety of endocrine functions and increase the risk for neoplasia. Epidemiological data reported increased prevalence of pituitary tumors in high industrial areas while genotyping studies showed that mutations in the aryl hydrocarbon receptor (AhR) interacting protein (AIP)—chaperone to the dioxin ligand AhR—gene are linked to predisposition to pituitary tumor development. Aim of the present study was to establish whether endocrine pollutants can induce cell proliferation in normal rat pituitary cells.

**Methods:**

Pituitary primary cultures were incubated with 250, 650 and 1250 pM benzene or 2-ethyl-phthalate for up to 96 h and viability, energy content and cell proliferation assessed. Expression of pituitary tumor transforming gene (*PTTG*), cyclin D1 (*Ccnd1*), *AhR* and *AIP* was quantified by RT-qPCR.

**Results:**

Incubation with benzene or 2-ethyl-phthalate increased viability and energy content in pituitary cells. The endocrine disruptors also increased cell proliferation as well as *Ccnd1* and *PTTG* expression. Increased *AhR* and *AIP* expression was observed after incubation with the two pollutants.

**Conclusions:**

Our findings indicate that benzene and 2-ethyl-phthalate activate *AhR/AIP* expression and stimulate proliferation in normal rat pituitary cells. This study is the first demonstration that pollutants can induce normal pituitary cells to proliferate and provides a link between epidemiological and genomic findings in pituitary tumors.

**Electronic supplementary material:**

The online version of this article (doi:10.1007/s11102-016-0777-3) contains supplementary material, which is available to authorized users.

## Introduction

Endocrine disruptors are widely distributed chemical pollutants known to affect endocrine functions [1, 2], in particular reproduction and development. Indeed, it is known since the early 1970s that breeding patterns, sex of offspring and fetal maturation are variably affected by endocrine toxicants [3, 4] as is hormonal production [2, 5].

More recently, the carcinogenic potential of endocrine disruptors has become a major research focus following epidemiological data showing an association between endocrine disruptor exposure and breast, prostate, testis and thyroid neoplasia [6–10]. In support of this evidence, in vitro studies showed that endocrine disruptors induce cell cycle deregulation, death and proliferation in breast and ovarian cancer cell lines [11–13]. Similar growth-promoting effects have also been reported for estrogen-sensitive pituitary adenoma cell lines, e.g. MtT/E2 [14, 15], GH_3_ [16, 17], suggesting that endocrine disruptors may be linked to pituitary tumor development. Further, in vivo models revealed a higher incidence of pituitary adenomas in rats treated with a mixture of endocrine disruptors [18]. In humans, epidemiological studies showed an increased prevalence of growth hormone (GH)-secreting pituitary tumors in high industrial density areas [19] and, possibly, higher incidence of pituitary neoplasia following the accidental spillage of dioxin [20]. An additional link between endocrine disruptors and pituitary tumorigenesis was provided by the discovery of mutations in the aryl hydrocarbon receptor interacting protein (*AIP*) gene in patients with pituitary tumors [21], as the aryl hydrocarbon receptor (AhR) is well-known to bind toxins and phytochemicals [22]. Indeed, the AhR pathway is called into play by several endocrine disruptors [23], both in the pituitary and in other tissues [24, 25].

Given this evidence, we decided to study whether endocrine disruptors affect normal rat pituitaries in vitro. Our findings indicate that long-term incubation with benzene and 2-ethyl-phthalate increases cell viability, energy content and proliferation in normal rat pituitary cells. Further, we observed an increase in genes associated with cell cycle progression and pituitary tumorigenesis as well as in *AhR* and *AIP* expression. Taken together, our findings show for the first time that endocrine pollutants can induce proliferation in normal pituitary cells and support the contention that endocrine disruptors play a role in pituitary tumorigenesis.

## Materials and methods

### Pituitary primary cultures

Rat anterior pituitaries were dissected from adult male Sprague–Dawley rats, sacrificed in accordance with animal care guidelines (National Institutes of Health, Office of Animal Cure and Use). The study was approved by the Ethical Committee of the Grant Coordinating Institution, i.e. University of Messina, Italy. Pituitaries were cultured using our usual protocol [26, 27]. Briefly, pituitaries were trypsin-digested and dispersed cells plated at 50,000 cells/well in 96 multi-well plates for cell assays and at 50,000 cells/well in 24 multi-well plates for RT-qPCR. Wells were incubated in Dulbecco’s modified medium (DMEM), 10% fetal bovine serum (FBS), antibiotics for 3–4 days (Sigma, Saint Louis MO, USA) prior to experimental procedures.

### Treatments

After 3–4 days attachment, cells were washed for 1 h in Dulbecco’s modified Eagle medium (DMEM) and 0.1% bovine serum albumin (BSA) then treated with 250, 650, 1250 pM benzene (Sigma, Saint Louis MO, USA) or bis-(2-ethylhexyl)-phthalate (2-ethyl-phthalate; Sigma, Saint Louis MO, USA) for 3, 24 or 96 h. Wells were examined by light microscope prior and at the end of incubations in order to exclude fibroblast contamination; experience over the past 20 years showed that contamination with fibroblasts or stromal cells does not constitute a problem with the current cell dispersion protocol. Incubation with 250 µg/ml cycloheximide (CHX) (Sigma, Saint Louis MO, USA) served as representative control for cytotoxicity given that high doses of the protein synthesis inhibitor have been shown to be cytotoxic [28, 29]; wells incubated with DMEM + 0.1% BSA represented untreated control. Treatments were repeated in four separate experiments on quadruplicate wells.

### Cell assays

Metabolic cell energy content was measured by ATP lite (Perkin Elmer, Waltham MA, USA) according to the manufacturer’s instructions. Wells were incubated in ATPlite assay reagent at room temperature and luminescence assessed after 10 min.

Cell viability was measured by methylthiotetrazole (MTT) assay (Sigma, Saint Louis MO, USA). MTT was added to wells and cells incubated at 37 °C for 3 h. Medium was subsequently discarded and cells dissolved in 1:25 1 N HCl/100% propanol. Absorbance was read at 540 nm.

Apoptosis was tested by Caspase Glo 3–7 assay (Promega, Madison WI, USA). Wells were incubated in Caspase 3–7 reagent at room temperature and luminescence assessed after 30 min.

Proliferation was assessed by 5-bromo-2′-deoxyuridine labeling (BrdU-labeling; Roche, Mannheim, Germany). Cells were incubated with BrdU-labeling reagent for 16 h, denatured then treated with anti-BrdU-POD antibody for 120 min. Substrate reaction solution was added and reaction stopped after 30 min with 1 M H_2_SO_4_. Colorimetric signal was measured at 450 nM.

### RNA extraction and RT-qPCR

RNA was extracted from pituitary primary cultures with Pure link RNA mini Kit (Life Technologies, Carlsbad CA, USA) and reverse-transcribed with SuperScriptR VILO™ cDNA Synthesis Kit (Invitrogen Life Technologies, Carlsbad CA, USA). Quantitative Real-Time PCR (qRT-PCR) for Cyclin D (*Ccnd1*) and pituitary transforming gene 1 (*Pttg1*) was performed using Platinum Quantitative PCR Supermix-UDG with premixed ROX Taqman assay (Applied Biosystem, Foster City CA, USA) for the detection of *Ccnd1* probe Rn00432360_m1 and *Pttg1* probe Rn00574373_m1 with hypoxanthine–guanine phosphoribosyltransferase *(Hprt1;* probe Rn01527840_m1) as endogenous control on a 7900 HT sequence Detection System (Applied Biosystem, Foster City CA, USA). For *AhR* and *AIP* expression, primers were designed using Beacon Designer 5.0 software (see Online Resource ESM1.pdf) and qRT-PCR performed using BioRad MiIQ Detection System (BioRad Laboratories, Hercules CA, USA) with SYBR green fluorophore. A melting curve analysis was performed following every run to ensure a single amplified product. Basal expression data (2^−ΔCt^) was calculated and normalized to house-keeping genes (Online Resource ESM1.pdf); expression after treatment was analyzed as 2^−ΔΔCt^ and expressed in fold increase.

### Statistical analysis

Kruskal–Wallis test was used for comparisons between treatments (Statview 5.0, Cary NC, USA) and *p* < 0.05 considered statistically significant. Treatment values are given relative to control and expressed as mean ± S.E.M.

## Results

### Exposure to benzene and 2-ethyl-phthalate modulates cell metabolism, viability and proliferation

**Fig. 1:**
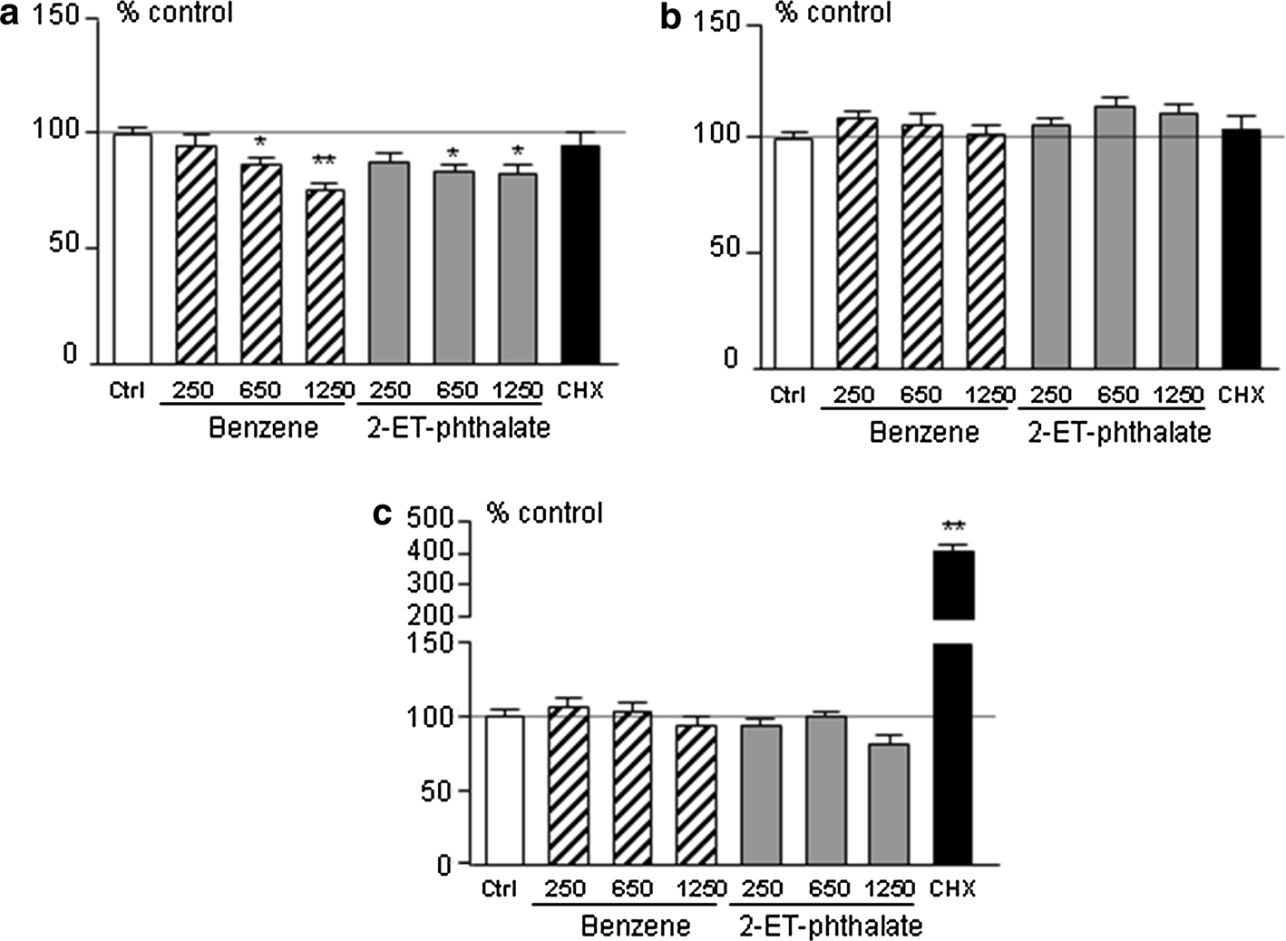
Cell energy content (**a**), viability (**b**) and apoptosis (**c**) in rat anterior pituitary primary cultures treated with 250, 650, 1250 pM benzene (Ben, *striped bars*) or bis-(2-ethylhexyl)-phthalate (2-ET, *grey bars*), 250 µg/ml of cycloheximide (CHX, *black bar*) for 24 h. *White bars* represent control wells treated with plain medium (Ctrl). Data were normalized to control values and expressed as percentage of control; *bars* represent mean ± SEM from four separate experiments

Short-term incubation, i.e. 3 h, with benzene and 2-ethyl-phthalate did not affect cell metabolism, cell viability or apoptosis in rat anterior pituitary primary cultures (see Online Resource ESM2.pdf). Conversely, 24-h incubation with benzene and 2-ethyl-phthalate decreased ATP levels (Fig. 1a), attesting to decreased intracellular energy at this time point. This was not associated with cell death, as no induction of apoptosis could be observed (Fig. 1c). On the other hand, cell viability at 24-h exhibited a slight, not significant, increase (Fig. 1b).

**Fig. 2:**
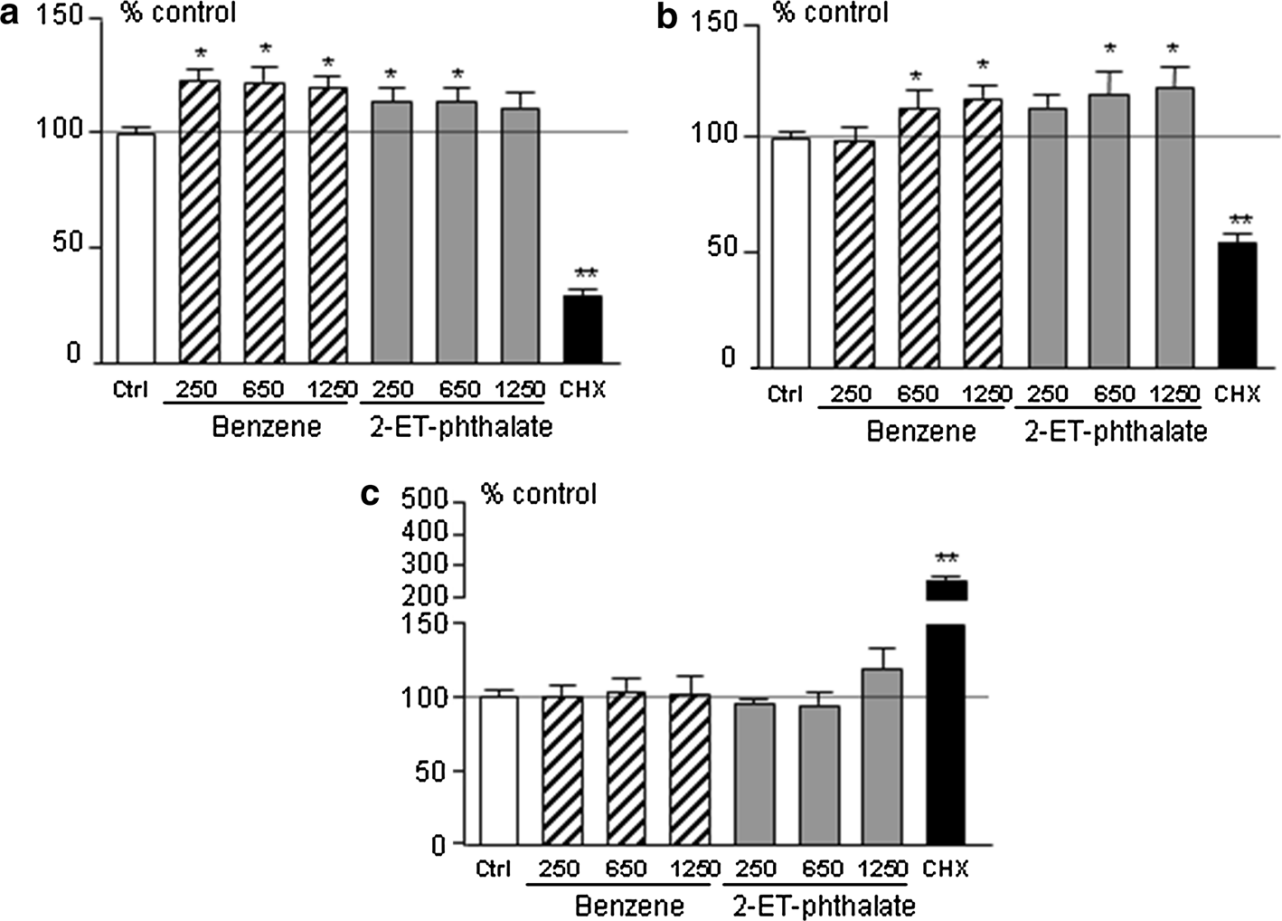
Cell energy content (**a**), viability (**b**) and apoptosis (**c**) in rat anterior pituitary primary cultures treated with 250, 650, 1250 pM benzene (Ben, *striped bars*) or 2-ethyl-phthalate (2-ET, *grey bars*), 250 µg/ml of cycloheximide (CHX, *black bar*) for 96 h. *White bars* represent control wells treated with plain medium (Ctrl). Data were normalized to control values and expressed as percentage of control; *bars* represent mean ± SEM from four separate experiments

**Fig. 3:**
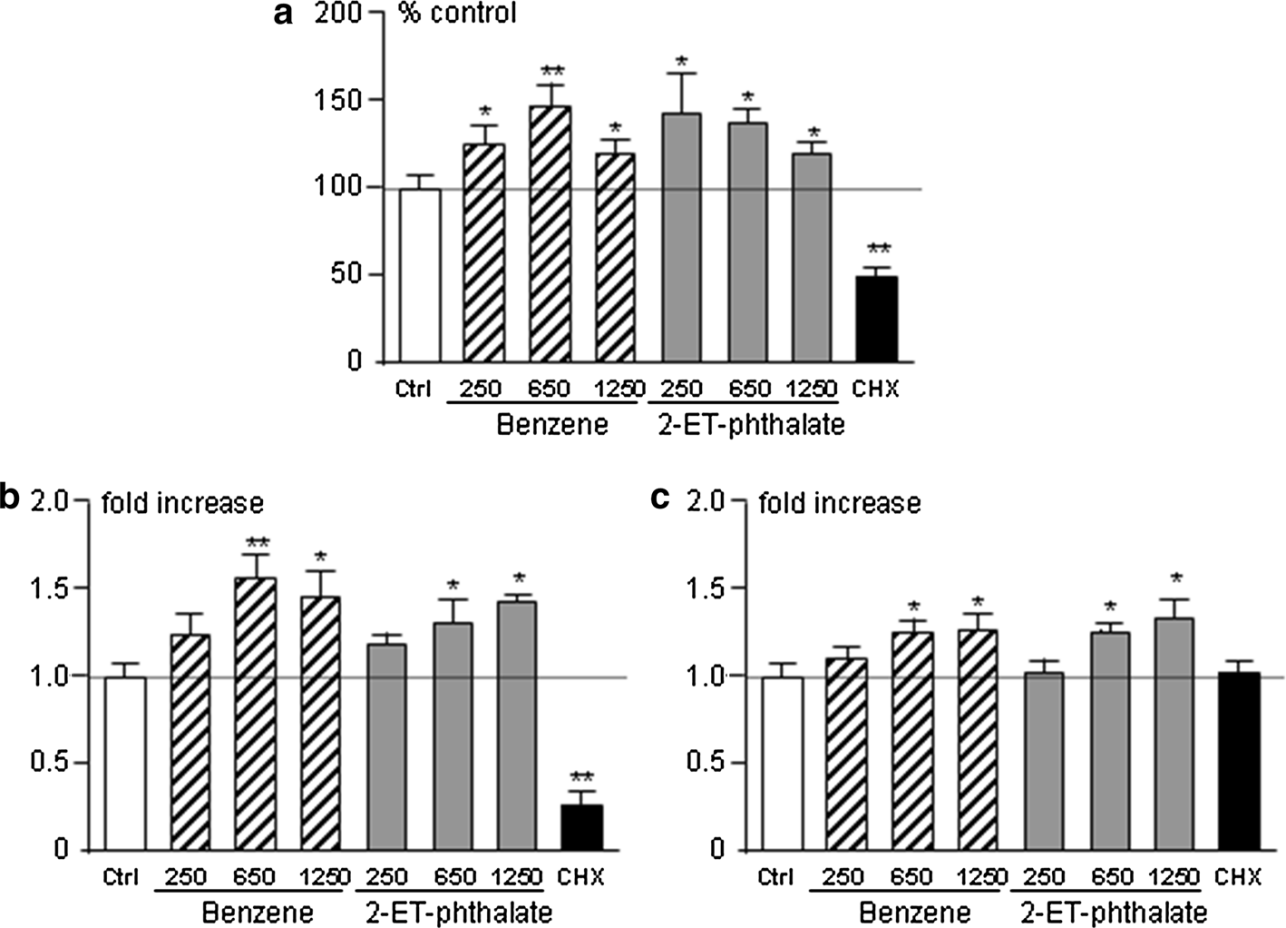
Cell proliferation (**a**) and *Ccnd1* (**b**) and *Pttg1* expression (**c**) in rat anterior pituitary primary cultures. Cells were treated with 250, 650 pM benzene (Ben, *striped bars*) or 2-ethyl-phthalate (2-ET, *grey bars*), 250 µg/ml of cycloheximide (CHX, *black bar*) in proliferation experiments and with 650, 1250 pM benzene (Ben, *striped bars*) or 2-ethyl-phthalate (2-ET, *grey bars*) for mRNA quantification experiments. Both experiments were carried out for 96 h. *White bars* represent control wells treated with plain medium (Ctrl). Data were normalized to control values and expressed as percentage of control in proliferation experiments and fold-increase in gene expression experiments; *bars* represent mean ± SEM from three separate experiments

Given these results, we tested the effect of prolonged, e.g. 96 h, incubation on rat anterior pituitary primary cultures and observed an increase in cell energy content (Fig. 2a) as well as increased cell viability (Fig. 2b). We therefore decided to assess proliferation by Brd-U incorporation and Cyclin D1 expression, a marker of cell cycle progression. The percentage of Brd-U positive cells was increased in wells treated with benzene or 2-ethyl-phthalate compared to control wells (Fig. 3a) as was *Ccnd1* expression (Fig. 3b), attesting to increased proliferation of rat anterior pituitary cells after 96-h incubation. In view of these effects, we evaluated expression of pituitary tumor transforming gene (PTTG), a protooncogene implicated in pituitary tumorigenesis [30], and, indeed, could observe an increase in *Pttg1* expression in wells treated with benzene and 2-ethyl-phthalate (Fig. 3c). As expected, cyclohexamide reduced cell metabolism, viability and proliferation and induced cell apoptosis (Figs. 2, 3).

### Exposure to benzene and 2-ethyl-phthalate increases AhR/AIP expression

**Fig. 4:**
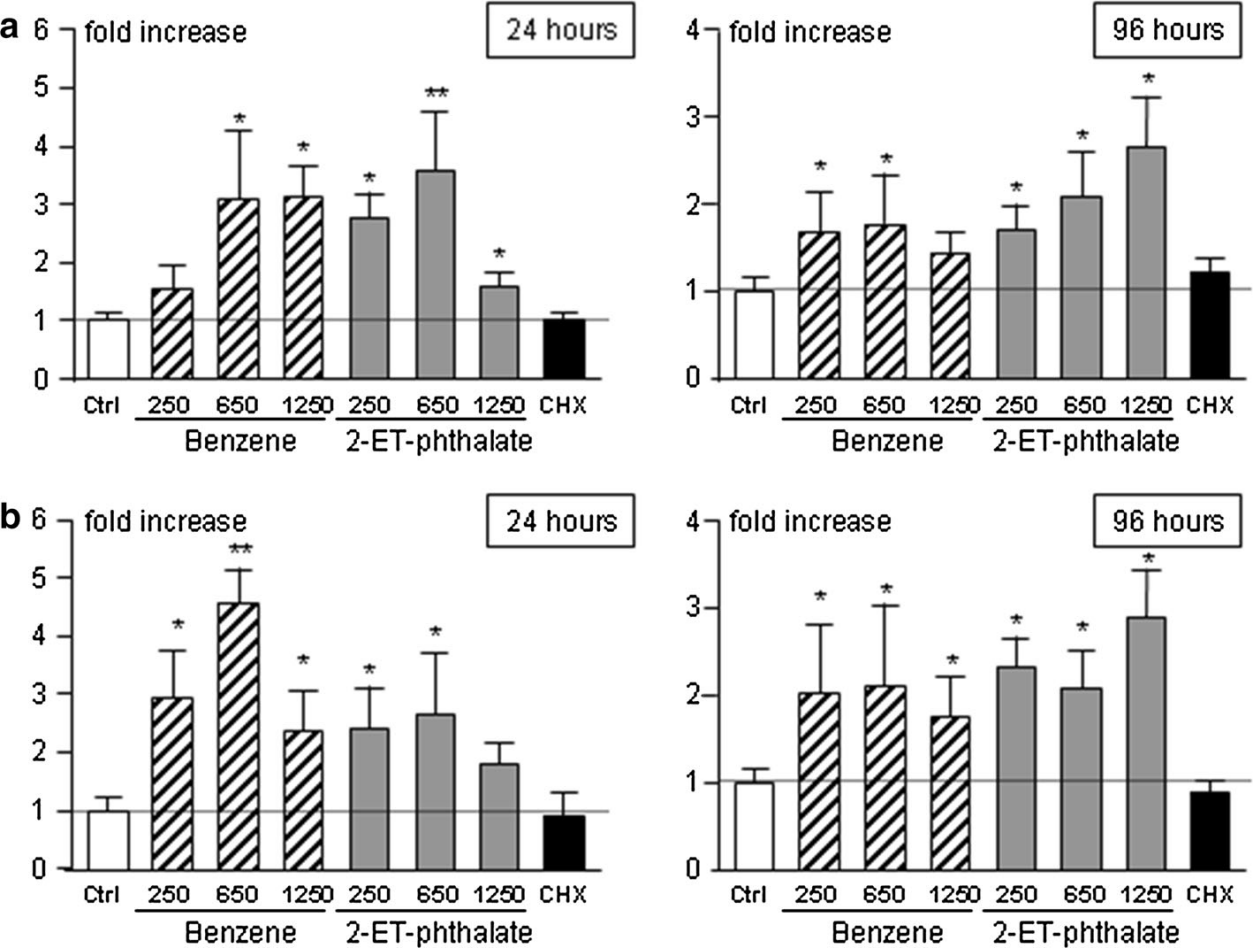
Quantification of *AhR* (**a**) and *AIP* (**b**) expression in rat anterior pituitary primary cultures treated with 250, 650, 1250 pM benzene (Ben, *striped bars*) or 2-ethyl-phthalate (2-ET, *grey bars*), 250 µg/ml of cycloheximide (CHX, *black bar*) for 3, 24 and 96 h. *White bars* represent control wells treated with plain medium (Ctrl). Expression data were analyzed as 2^−∆∆Ct^ in three independent experiments and expressed as fold increase over control

Given the role of AhR as a mediator of endocrine disruptors [23] and of AIP, its chaperone protein, in pituitary tumorigenesis [21] we decided to study whether incubation with benzene or 2-ethyl-phthalate affects expression of either gene. No effect of the two endocrine disruptors were observed after 3 h whereas a clear-cut increase in both *AhrR* and *AIP* expression was apparent after 24- and 96-h incubation (Fig. 4).

## Discussion

Our results show that benzene and 2-ethyl-phthalate stimulate rat anterior pituitary cell proliferation, an important finding given the increasing evidence of endocrine-disruptor induced tumorigenesis [3]. In fact, although endocrine disruptors were initially discovered due their adverse effect on reproduction and fetal development, subsequent studies demonstrated a role in an variety of endocrine disorders and, eventually, endocrine-related cancers [4, 31]. The mechanisms underlying endocrine disruptor-induced carcinogenesis are varied and as yet not fully understood but appear to comprise receptor agonism or antagonism, activation of oncogenes and/or repression of tumor suppressor genes, changes in intracellular signaling pathways and DNA methylation patterns. These effects are associated with alterations ranging from hyperplasia to carcinoma [32–34], increased risk of cancer and, ultimately, increased cancer-related mortality [3, 10].

Pituitary tumors are common intracranial neoplasias with site-related symptoms and systemic morbidity due to hormonal excess. Most are sporadic, slow-growing and diagnosed in middle-aged to older individuals [35]. Epidemiological studies are few but recent reports of increased prevalence of pituitary adenomas in high industrialized areas [19] and, possibly, after toxic spillage [20] suggested a link to environmental causes.

Experimental data provided support for this association as endocrine pollutants have been shown to exert several effects in cell lines derived from rat pituitary neoplasms, most notably estrogen-sensitive somatotropes, i.e. GH_3_, and mammosomatotropes, i.e. MtT/E-2. Bisphenol A, genistein, o,p′-DDT, cadmium and endosulfan have all been shown to increase proliferation in either cell line [14–17, 36]. Further, increased GH and prolactin synthesis and release has been observed with toxaphene, bisphenol A, dioxin and other alkyl-phenols [37–40].

Our study shows for the first time that endocrine pollutants can stimulate proliferation in normal adult pituitary cells. Our finding is of particular relevance given that the abovementioned studies have been performed on tumoral pituitary cell lines, thus unsuitable to study the development of pituitary tumors. So far, only cadmium, a heavy metal with long half-life and estrogen-like activity, has been studied in the normal pituitary in vitro and increased cell growth was observed after 96-h incubation [36]. In this context, it has been reported that perinatal administration of endocrine disruptors is associated with increased incidence of pituitary tumors in grown rats [18] and, interestingly, that cats with acromegaly present higher plasma concentrations of halogenated contaminants, such as polychlorinated biphenyls, polybrominated diphenyl ethers and dichlorophenyl ethane, compared to non-acromegalic cats [41]. Altogether, it appears that the normal pituitary is indeed sensitive to the proliferative effect of endocrine contaminants.

Endocrine disruptors were first identified as compounds with estrogenic potential [42] and, as such, act via the estrogen/androgen receptor pathway [43, 44]. Indeed, the estrogen receptor is involved also in stimulation of proliferation and transcription in pituitary cell lines [14, 36, 39, 45], mainly via the ERK pathway [16]. Current evidence demonstrates that disruptors call several additional pathways into play including the AhR–AIP–ARNT system [23]. AhR is a cytosolic transcription factor first identified through its dioxin-binding capacity and, indeed, mediates a variety of responses to toxic halogenated aromatic hydrocarbons [22]. AIP acts as chaperone to AhR and facilitates activation of AhR; in turn, activated AhR translocates into the nucleus, heterodimerizes with AhR-nuclear translocator (ARNT) and acts upon target genes [46]. The role of this pathway in carcinogenesis is the focus of increasing interest [23] and, indeed, a link to pituitary tumorigenesis was recently detected as germline mutations in *AIP* were shown to predispose to development of pituitary adenomas [21]. Several studies followed upon this first report in an attempt to clarify the pathogenesis of *AIP*-mutated pituitary tumors but the exact mechanism remains elusive [47, 48]. In fact, expression and cellular localization of AIP, AhR and ARNT appear variable with some tumors presenting low *AIP*, absent nuclear AhR staining and loss of *ARNT* expression, others increased *AIP* expression or nuclear AhR staining [49–51]. A most recent study in fibroblasts from patients with four different *AIP* mutations showed that *AhR* expression was unaffected but that AhR target genes, i.e. *CYP1B1*, AhR repressor (*AHRR*), were either reduced or increased depending on the *AIP* variant [52]. From a clinical viewpoint, patients carrying *AIP* mutations are more often young, male and with large GH- or mixed GH- and prolactin-secreting tumors [48, 53, 54]. Interestingly, the *AhR* gene itself appears to contribute to severity of acromegaly as polymorphisms and variants in *AhR* have been associated with more aggressive disease [55, 56].

Altogether, it is clear that the AhR–AIP pathway is involved in pituitary tumorigenesis and our findings shed further light into this concept. We observed an increase in *AhR* and *AIP* expression during treatment with benzene and 2-ethyl-phthalate, which represents the first evidence for upregulation of *AhR/AIP* gene expression in normal pituitaries in vitro. Data in pituitary cell lines showed that some AhR ligands, e.g. ß-naphtoflavone, prothioconazole, reduce AhR expression [57] and function [24] while other AhR ligands, e.g. 2-methyl-4-chlorophenoxyacetic acid, tau-fluvalinate, stimulate AhR activity [24]. In contrast, dioxin, the main AhR ligand, failed to affect AhR expression in pituitary cells in vitro [58] and no changes in anterior pituitary *AhR* expression up to 4 weeks after dioxin administration were observed in vivo [59]. As regards benzene and 2-ethyl-phthalate, both act via AhR in different cell models [60, 61] and our evidence now shows that these endocrine disruptors modulate the pituitary AhR/AIP pathway.

Last, one word of comment on our research protocol. As mentioned above, our study evaluated the effects of two specific pollutants, benzene and 2-ethyl-phthalate, rather than a mixture of pollutants, as usually occurs for both routine and occupational exposure. Indeed, research strategies into the impact of endocrine disruptors encourage testing with a variety of chemicals at different dosages in distinct stages of development [62]. In order for this research to prove significant, however, there has to be some evidence on the effect of one or another pollutant in a given tissue. Our findings prove that benzene and 2-ethyl-phthalate stimulate proliferation in adult rat pituitary cells and provide the basis for further studies aimed at expanding upon our results, e.g. susceptibility in adult vs early life, effect of low-dose chemical mixtures, multigenerational studies in exposed areas [3].

## Electronic supplementary material

Below is the link to the electronic supplementary material.

Supplementary material 1 (PDF 9 kb)

Supplementary material 2 (PDF 111 kb)
