# Supplementary material for: Benzene and 2-ethyl-phthalate induce proliferation in normal rat pituitary cells
Source: Pituitary. 2016 Nov 16;20(3):311–8. doi: 10.1007/s11102-016-0777-3 (PMC5427103; doi:10.1007/s11102-016-0777-3)
Supplement: Supplementary file 1 — Supplementary material 1 (PDF 9 kb) [file 11102_2016_777_MOESM1_ESM.pdf]

Supplemental Table 1- Primers for *AIP* and *AhR* qRT-PCR

**Primers**		**Sequences (5'-3')**
AIP	sense	TCGTGCGTACCATGCGTGAG
	antisense	TGTTGCGGAGGCTCTTGGC
AhR	sense	ACCAGTGTAGAGCACAAGTCAGAG
	antisense	AGACGCATAGAAGACCAAGGCATC
ßACT	sense	CCACACCCGCCACCAGTTC
	antisense	GACCCATACCCACCATCACACC
ßmicroglobulin	sense	TCTTTCTGGTGCTTGTCTCTCTGG
	antisense	CTATCTGAGGTGGGTGGAACTGAG
L13A	sense	AGGTGGTGGTTGTACGCTGTG
	antisense	GGTTGGTGTTCATCCGCTTTCG
