# Supplementary material for: Benzene and 2-ethyl-phthalate induce proliferation in normal rat pituitary cells
Source: Pituitary. 2016 Nov 16;20(3):311–8. doi: 10.1007/s11102-016-0777-3 (PMC5427103; doi:10.1007/s11102-016-0777-3)
Supplement: Supplementary file 2 — Supplementary material 2 (PDF 111 kb) [file 11102_2016_777_MOESM2_ESM.pdf]

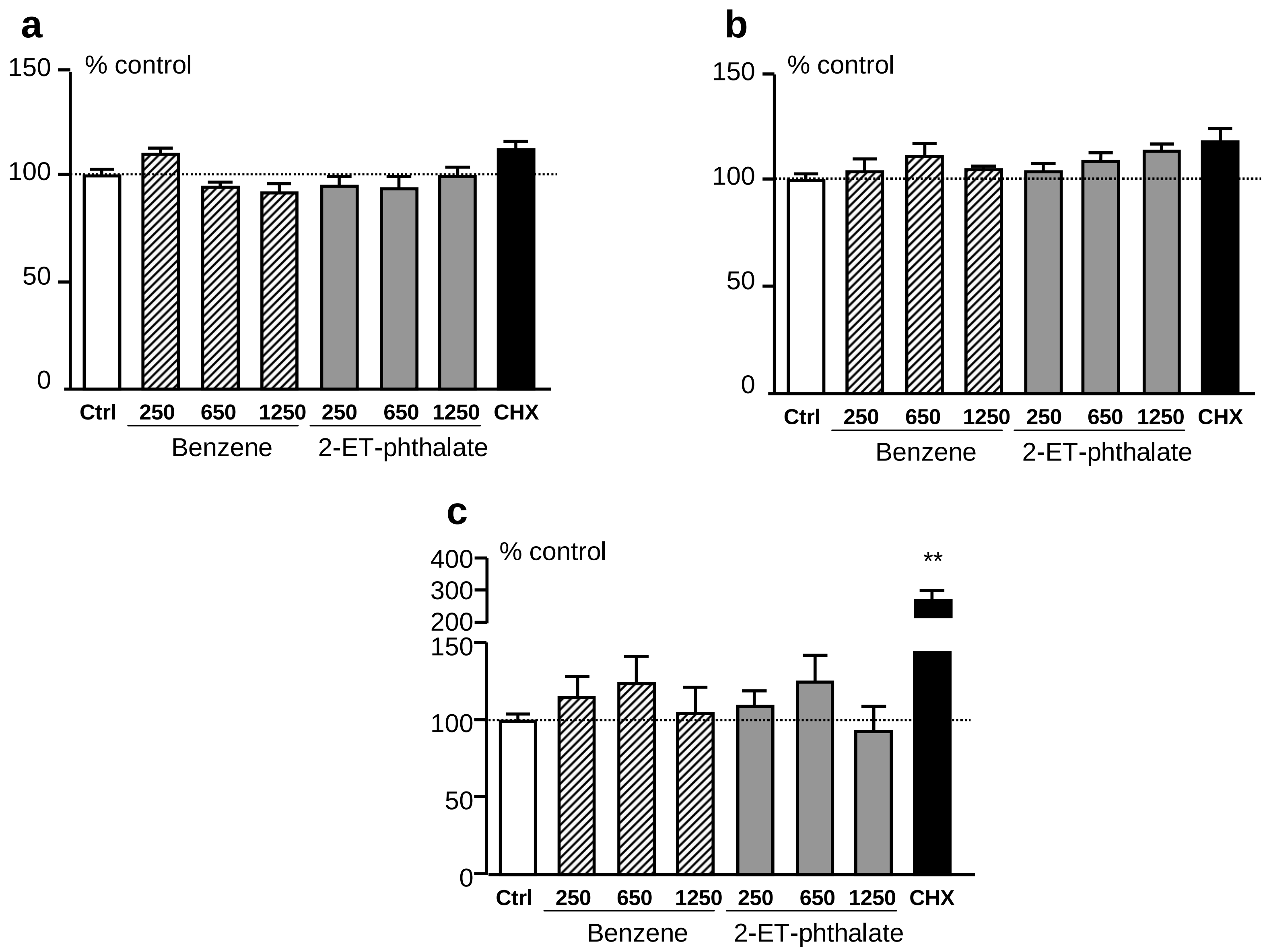

Cell energy content (**a**), viability (**B**) and apoptosis (**C**) in rat anterior pituitary primary cultures treated with 250 pM, 650 pM, 1250 pM benzene (Ben, striped bars) or bis-(2-ethylhexyl)-phthalate (2-ET, grey bars), 250 µg/ml of cycloheximide (CHX, black bar) for 3 hours. White bars represent control wells treated with plain medium (Ctrl). Data were normalized to control values and expressed as percentage of control; bars represent mean ± SEM from 4 separate experiments.
